# Pathogenesis of cerebral malaria: new diagnostic tools, biomarkers, and therapeutic approaches

**DOI:** 10.3389/fcimb.2015.00075

**Published:** 2015-10-27

**Authors:** Praveen K. Sahu, Sanghamitra Satpathi, Prativa K. Behera, Saroj K. Mishra, Sanjib Mohanty, Samuel Crocodile Wassmer

**Affiliations:** ^1^Center for the Study of Complex Malaria in India, Ispat General HospitalRourkela, India; ^2^Department of Pathology, Ispat General HospitalRourkela, India; ^3^Department of Microbiology, New York University School of MedicineNew York, NY, USA; ^4^Department of Pathology, The University of SydneySydney, NSW, Australia

**Keywords:** cerebral malaria, *Plasmodium falciparum*, diagnostic, pathophysiology, research tools, new therapies

## Abstract

Cerebral malaria is a severe neuropathological complication of *Plasmodium falciparum* infection. It results in high mortality and post-recovery neuro-cognitive disorders in children, even after appropriate treatment with effective anti-parasitic drugs. While the complete landscape of the pathogenesis of cerebral malaria still remains to be elucidated, numerous innovative approaches have been developed in recent years in order to improve the early detection of this neurological syndrome and, subsequently, the clinical care of affected patients. In this review, we briefly summarize the current understanding of cerebral malaria pathogenesis, compile the array of new biomarkers and tools available for diagnosis and research, and describe the emerging therapeutic approaches to tackle this pathology effectively.

## Cerebral malaria: a complex and multi-factorial syndrome

*Plasmodium falciparum* malaria continues to be the predominant infectious disease in tropical and sub-tropical countries, with an estimated global incidence of 207 million cases and 627,000 deaths reported in 2012 (W.H.O., 2014). Cerebral malaria (CM) is a severe complication of *P. falciparum* infection. This complex and potentially reversible encephalopathy leads to coma and occurs with or without signs of compromise in other organs. There is no definite adjunctive therapy and even with highly effective antimalarial drugs and intensive care, mortality is 10–25% (Taylor et al., 2004; Mishra and Newton, 2009). CM is most frequent in sub-Saharan Africa where the intense malaria transmission leads to widespread acquisition of immunity during childhood. Thus, CM is rare in adults and principally occurs in children under five (W.H.O., 2014). CM is also an important cause of mortality and morbidity in South East Asia, where malaria transmission is not sufficiently intense to induce robust immunity. In this region, CM principally occurs in older children and adults.

There are significant differences in the pattern of vital organ dysfunction in CM between African children and South East Asian adults (Trang et al., 1992; Marsh et al., 1995; Newton et al., 1998; Wassmer et al., 2015) for which the mechanism is poorly understood. In adults, central nervous system dysfunction frequently occurs in conjunction with failure of other organ systems, particularly renal and respiratory. In contrast, African children have a more purely neurological disease, with rapid onset of coma, anemia and seizures, but overt respiratory or renal compromise is generally absent (Newton et al., 1998; Miller et al., 2013). Neuropathological dissimilarities were also described: African children present prominent accretions of fibrin (Dorovini-Zis et al., 2011; Moxon et al., 2013; Milner et al., 2014), platelets (Grau et al., 2003), and inflammatory infiltrates within the neurovasculature. These features are less marked or absent in Southeast Asian adults (Macpherson et al., 1985; Turner et al., 1994; Hawkes et al., 2013).

Several processes have been implicated in CM pathogenesis, including microvascular obstruction by *P. falciparum*-parasitized red blood cell (PRBC; Berendt et al., 1994), excessive pro-inflammatory cytokine production (Clark and Rockett, 1994), microvascular thrombosis (van der Heyde et al., 2006; Moxon et al., 2009), loss of endothelial barrier function (Beare et al., 2009; Dorovini-Zis et al., 2011), and endothelial dysregulation (Wassmer et al., 2011). The way these pathological mechanisms are linked and how they are influenced by host and parasite factors remains to be elucidated. In addition, the reasons why circulating cytokines, coagulation factors, or PRBC specifically target only the brain in African children, and the brain as well as other organs in Southeast Asian adults, are still unclear.

In order to better understand the factors leading to the development of CM and subsequently improve the outcome for affected patients, a new range of techniques and sets of biomarkers for severity have recently been developed. The present review focuses on these innovative advances, which offer a new panel of tools for malaria researchers and clinicians in the field.

## Novel investigative techniques

### Clinical neuroimaging

Advanced imaging devices have become increasingly accessible to malaria-endemic countries in recent years, allowing a leap forward in terms of clinical studies aimed at elucidating the etiology of CM.

#### Magnetic resonance imaging

MRI yields very useful clinical information and provides a measure of several neurological parameters impossible to assess otherwise, e.g., nature of cerebral blood flow alteration and damage to neural tissue. The study by Penet et al. (2005) was the first to perform MRI of the murine model of CM, and demonstrated vascular damage attributable to inflammatory processes. Subsequently, MRI techniques also allowed the analysis of neuronal axon injury during CM (Kennan et al., 2005), as well as the investigation of the diffused cerebral swelling of brainstem in 120 Malawian children with CM (Potchen et al., 2012). By allowing the comparison of specific parameters between CM patients who survive and those who succumb to the disease, the use of MRI has been instrumental in highlighting an increased intracranial pressure and brain stem herniation in fatal cases (Seydel et al., 2015). This study not only suggested for the first time a cause of death in pediatric CM, but also new potential therapeutic approaches aimed at decreasing the intracranial pressure in affected patients.

#### Computed tomography

Unlike MRI, CT scans are now commonly available in malaria-endemic countries. Their pioneering use in CM patients from Thailand and Kenya suggested for the first time the involvement of cerebral edema in the development of the pathology (Looareesuwan et al., 1983; Newton et al., 1994). Subsequently, a study in India showed that CT findings correlate well with level of consciousness and severity of disease but do not reveal the extent of the pathology permitted by *post-mortem* examinations (Patankar et al., 2002). CT imaging is particularly helpful for the determination of cerebral volume variation and the detection of infarctions in large vessels, as recently demonstrated in a pediatric CM patient population (Potchen et al., 2010). In children with retinopathy-confirmed CM, acute head CTs revealed findings consistent with autopsy studies and showed abnormality in basal ganglia, white matter and corpus callosum. Follow-up images in survivors allowed the identification of lesions consistent with acute symptomatology and chronic deficits. CT imaging was used in a consequent study to evaluate and map brain swelling in Indian adults with CM and assess the potential benefits of mannitol as an adjunct therapeutic agent (Mohanty et al., 2011). The study was the largest of its kind, including 126 Asian adult patients with CM. Their systematic acute head CT scans revealed that 29% had moderate to severe brain swelling. There was, however, no significant correlation between swelling and coma depth and mortality in the series.

### Investigative neuro-imaging tools

While the use of neuroimaging techniques has contributed to a better understanding of the pathophysiology of CM, novel and revolutionary approaches have become available in the laboratory but are not applicable directly to patients. For this, the experimental model, albeit limited (Craig et al., 2012), represents a useful tool to investigate the pathogenesis of CM at the cellular and molecular level in the brain.

#### *In vivo* bioluminescent imaging

*In vivo* bioluminescent imaging is a versatile and sensitive tool that is based on the detection of light emission from cells or tissues. The technique has been allowed by the genetic modification of malaria parasites and the production of luciferase-expressing lines. This, coupled with the development of imaging systems to detect cells expressing reporter genes, has significantly broadened the possibilities for *in vivo* studies of interactions between *Plasmodium* spp. parasites and their hosts (Franke-Fayard et al., 2006). Optical imaging by bioluminescence allows a low-cost, non-invasive and real-time analysis of disease processes at the molecular level in experimental animals. It also permits longitudinal monitoring of the course of the pathology in the same animal, and the imaging of transgenic fluorescent or bioluminescent malaria parasites is now widely used as a tool to assess parasite distribution during experimental CM. A recent study used real-time *in vivo* imaging to evaluate the contribution of different immune mediators to PRBC accumulation and distribution during the development of experimental CM. The results showed that CD8+ T cells and IFN-γ are responsible for the rapid increase in total parasite biomass, as well as for the accumulation of PRBC in the brain and in different organs (Claser et al., 2011). These *in vivo* pathogenesis studies can also be carried out with a different bioluminescent target, as shown by Imai and colleagues, who evaluated oxidative stress during experimental CM using OKD48 (Keap1-dependent Oxidative stress Detector, No-48-luciferase) mice. Oxidative stress in the brain can be visualized in these animals after injection of luciferin, and an elevated bioluminescent signal was associated with the development of the pathology (Imai et al., 2014). Lastly, this imaging technique can also used for the assessment of parasite virulence (Spaccapelo et al., 2010) and provides a simple and reliable framework for *in vivo* antimalarial and CM adjunctive treatment screening by monitoring post-treatment changes in bioluminescence signal, which correlates with the degree of parasitemia in the animal (Franke-Fayard et al., 2008).

#### Intra-vital microscopy

Recently developed, this advanced imaging tool allows the direct and live visualization of the brain via a cranial opening (Volz, 2013). The technique can reveal cellular responses over time and space during the course of experimental CM and can be conducted under conditions closely approximating those of a natural environment. In addition, it presents the advantage of observing *in vivo* pathological events in the brain, including variations in hemodynamic events (Nacer et al., 2014) and vascular leakages (Frevert et al., 2014). By comparing the variation of these parameters between control and treated animal groups, intra-vital microscopy has allowed the assessment of intervention drugs, including nimodipine and nitric oxide (NO) therapy (Cabrales et al., 2011; Zanini et al., 2011; Rénia et al., 2012). It is a versatile platform, as demonstrated by two recent and innovative studies. First, Cabrales and colleagues performed the direct, quantitative, and dynamic analysis of fluctuations of oxygen transport and tension during experimental CM progression and its contribution to the severity of disease. Results highlighted the pial tissue as highly sensitive to changes in blood flow, anemia, and low oxygen tension impacting sufficient oxygen delivery (Cabrales et al., 2013). Second, Pai and colleagues used of a novel, two photon-based approach, which allowed them to monitor the behavior of leukocytes in cerebral microvessels during the development of the pathology in infected mice. A decrease in the rolling velocity of monocytes, a measure of endothelial cell activation, was associated with the progressive worsening of signs in the animals. These modifications were mediated by *Plasmodium*-specific CD8+ T lymphocytes, suggesting their direct influence in the regulation of vascular pathology associated with the development of experimental CM (Pai et al., 2014).

#### ^18^F-fluorodeoxyglucose (FDG) positron emission tomography (PET)

FDG-PET is a non-invasive imaging tool used to map cerebral metabolic activity by quantifying the uptake of a glucose analog by brain cells. This metabolic activity was measured *in vivo* during the progression of experimental CM in the *Plasmodium coatneyi* primate model of the pathology (in which there is significant cerebral sequestration). The analysis revealed diffuse and heterogeneous reduction of metabolism in the cortex during the acute phase of infection (Sugiyama et al., 2004). These results are consistent with a focal impairment of the microcirculation, potentially induced by PRBC sequestration. However, it is plausible that this reduced metabolic activity safeguards the cerebral tissue against hypoperfusion, as an inherent function of the microcirculatory system is to protect organs from the effects of diminished oxygen and metabolite supply (Ellis et al., 2005). This could explain why more than half of CM patients present no neurological sequelae following recovery (Kawai and Sugiyama, 2010). Another study showed reduced cerebral blood flow during CM using an FDG-PET in a murine model of experimental CM (Kennan et al., 2005). FDG-PET was recently used systematically in a cohort of patients to help the diagnosis of fever of unknown origin (Tokmak et al., 2014), showing that this approach might become available to CM patients soon and may be able to complement the ongoing MRI studies to shed some light on the pathophysiological processes during the neurological syndrome.

## New diagnostic tools

In addition to prevention strategies and effective treatment, one of the most important factors influencing the outcome of CM is its early diagnosis. According to a study published in 2004, about a quarter of the pediatric patients diagnosed with CM using the WHO criteria were shown at autopsy to have died of non-CM causes (Taylor et al., 2004), which highlights the importance of accurate and reliable diagnostic tools.

### Malarial retinopathy

The sequestration of *P. falciparum*-infected red blood cells (PRBC) in the cerebral microvasculature is the hallmark of CM. In pediatric patients, retinal microvessels have been shown to sustain damage comparable to the ones occurring in the brain, making them an easily observable surrogate marker to assess the severity of cerebral pathology during CM (Beare et al., 2006; Maude et al., 2009). In recent years, the approaches adopted to assess and document the retinal changes during CM have evolved rapidly and are now available for clinical studies in endemic areas.

#### Funduscopy

Funduscopy is a relatively low-cost and easy technique to assess the presence of retinopathies, which allows the accurate distinction between malaria and non-malaria coma in CM patients. Retinal changes include vessel color changes, white-centered hemorrhages, and peri- and extramacular whitening. The severity of these changes during *P. falciparum* infection correlates strongly with patient mortality, and the identification of markers associated with the presence of retinopathies and therefore, of CM, may allow the early detection of patients at risk (Kariuki et al., 2014; Maccormick et al., 2014).

#### Optical coherence tomography (OCT)

OCT is an *in vivo* imaging tool for the detection of retinal changes. This imaging technique allows optical-signal acquisition by which high-resolution cross-sectional images of the retina, optic nerve-head and even the thickness of the retinal nerve fiber layer can be acquired and both qualitative and quantitative evaluations can be made (Sakata et al., 2009). Despite its non-invasive nature and high-resolution output, the use of OCT in malarial retinopathy has been difficult to implement systematically so far due to its costs, as well as practical issues. Indeed, patients need to be seated upright for the retinal analysis, which is problematic for comatose CM patients in intensive care units. However, a case of *P. vivax* retinopathy has been recently described using OCT (Lee et al., 2010), showing that the new development in high-resolution and high-speed OCT, along with the improvement in portability (Huang and Hirose, 2012) might make the technology extremely valuable for retinopathy analyses in malaria-endemic areas.

#### Teleophthalmology

While the use of retinopathy has helped increasing the accuracy of diagnosis in African children and more recently in Asian adults (Sayeed et al., 2011), its use is still infrequent, as systematic funduscopy requires a trained ophthalmologist, as well as expensive equipment that is not always available in field clinics. This has led to the recent development of easy-to-handle and affordable retinal cameras (Maude et al., 2011), as well as the emergence of modified handheld portable devices such as smartphones. In addition to their telecommunication functions, the most recent models possess diagnostic-quality imaging facility that meet the criteria necessary for accurate fundus examination and rapid diagnosis of retinopathy (Kumar et al., 2012; Maamari et al., 2014). This revolutionary “teleophthalmology” can be performed using cheap 3D printed fittings where the built-in flash of the phone provides the light source, and an installed application allows the easy and rapid photo-documentation of retinal abnormalities in CM patients (Myung et al., 2014). Such devices can be operated by healthcare workers after minimal training and the saved images can be sent by SMS or email to an ophthalmologist for rapid diagnosis.

#### Fluorescein angiogram framework

In order to help accurtely establish the presence of retinopathy in CM, the automated analysis of the retinal vasculature has become an active research area in the field of medical imaging in the recent years, both for its diagnostic and prognostic significance (Beare et al., 2004). A prerequisite to this appoach is the automated detection of blood vessels, and the past decade has witnessed the rapid development of methods for retinal vessel segmentation (Fraz et al., 2012). A recent study has demonstrated the novel use of an automated segmentation approach in fluorescein angiography, to extract retina vessel images and build an analysis framework (Zhao et al., 2015). The latter includes four main components: vessel segmentation, analysis of vessel geometry, salient feature generation, and vessel classification. This automated analysis to classify retinal vessel abnormalities showed an overall sensitivity, specificity, and accuracy similar to the ones obtained using two direct observers. Coupling this new approach to the teleophthalmology described above would allow a direct diagnosis of CM without sending acquired pictures of the retina to an off-site ophthalmologist, leading to a faster treatment of the patient.

### Electroencephalography (EEG) and Micro-EEG

#### EEG

This non-invasive technique to record electrical impulses of the brain by measuring voltage fluctuations due to ionic current within the neural tissue has been used in CM patients since the early 1990s (Thumasupapong et al., 1995). Prolonged and multiple seizures complicate a high proportion of cases of CM and can damage brain tissue by aggravating hypoxia, hypoglycemia, and intracranial hypertension. In recent years, the use of EEG has allowed the detection of these delayed CM sequelae, including neurodisabilities such as *status epilepticus*. Acute and serial EEGs are especially important for identifying subclinical seizures. A study performed in Kenya revealed that in about 25% of the enrolled pediatric CM patients, coma is due to continuing subtle seizure activity which is likely to go undetected, but is responsive to anticonvulsant drugs (Crawley et al., 1996). Subsequent studies in Kenya and in Mali showed an increased prevalence of epilepsy in patients who survived CM (Carter et al., 2004; Ngoungou et al., 2006). The recent inclusion of retinopathy as a criteria for CM diagnostic in a study in Malawian children helped to improve the accuracy of the diagnosis in enrolled patients, and to identify children with pre-existing neurological injuries, predispositions to adverse neurological outcomes, or non-malarial causes of coma. In this carefully defined cohort, almost a third of retinopathy-positive CM survivors developed epilepsy or other neurobehavioral sequelae (Birbeck et al., 2010).

#### Micro-EEG

One major limitation of the EEG studies is the serial post-discharge follow-up assessments, which involve multiple patient visits to the hospital, or home-based visits by nurses. These are not always possible and often present a logistical hindrance to EEG studies in the field. However, a miniature version of the EEG equipment is now available as a portable headgear that can accommodate up to 32 electrodes and connects via Bluetooth technology to a small monitoring machine. The micro-EEG diagnostic accuracy of *status epilepticus* is comparable to that of standard EEG systems (Grant et al., 2014) and will greatly facilitate not only the recording of brain as an easy diagnostic tool (Omurtag and Fenton, 2012), but will also allow an easier continuous recording of the patient after discharge. Indeed, patients can leave the hospital with the device and come back once the recording period is over. The tools is expected to soon be used for rapid monitoring and imaging of neuropathological sequelae, as well as a standard routine assessment in comatose patients with CM, as is already initiated in a phase 1 and 2 clinical trial for levetiracetam, a medication to control the seizures associated with pediatric CM (http://www.clinicaltrials.gov/ct2/show/study/NCT01660672).

## Biomarkers

Biomarkers include tools and technologies that can facilitate the prediction, cause, diagnosis, progression, regression, or outcome of treatment of disease. For pathologies of the nervous system, there is a wide range of techniques used to gain information about the brain in both the healthy and diseased state. These involve measurements directly on biological media such as blood or cerebrospinal fluid (CSF) from the patients; or measurements via brain imaging, which do not involve direct sampling of tissue but measure changes in the composition or function of the nervous system. While biomarkers have been used to diagnose and prognosticate the progress and outcome of many chronic diseases, the field of malaria research only recently moved in the direction of actively identifying biomarkers that can accurately discriminate the severe forms of malaria, and in particular, CM. Such biomarkers, once identified, validated, and integrated into rapid diagnostic tests, could allow the accurate and early identification of CM patients and their subsequent referral to tertiary healthcare facilities for prompt intervention. Recent clinical studies have identified serological factors that have the potential of being biomarkers. Based on their function and stage(s) of usage, these can be classified as early screening and diagnosis biomarkers, as well as prognostic biomarkers (Table 1).

**Table 1:** **Pathogenesis of cerebral malaria: recent discoveries and potential biomarkers**.

**Biomarker**	**Abbreviations**	**Type/Site**	**Method**	**Indicative events**	**Study**
**HUMAN CEREBRAL MALARIA**					
von Willebrand factor (VWF), propeptide	VWF, VWF propeptide	Plasma	ELISA based assays	Indicates acute and excessive endothelial cell activation (Weibel–Palade exocytosis)	Hollestelle et al., 2006; Bridges et al., 2010
Erythropoietin	Epo	CSF	ELISA based assays	High levels are associated with a neuroprotective role in pediatric CM	Casals-Pascual et al., 2008
Angiopoetin-1, -2	ANG-1, ANG-2	Endothelial (whole blood levels)	ELISA based assays	ANG-1 and -2 levels from whole blood allow the accurate discrimination between cerebral, severe (non-cerebral) malaria and uncomplicated malaria	Conroy et al., 2009
		Endothelial (serum levels)	ELISA based assays	Low ANG-1 levels at presentation is indicative of a poor outcome; Serum ANG-1 levels are significantly decreased and ANG-2 levels increased in children with CM compared to uncomplicated malaria patients and healthy controls	Lovegrove et al., 2009
		Endothelial (plasma levels)	ELISA based assays	Low ANG-1 levels at admission and gradual increase with recovery are observed, suggesting ANG-1 as a potential marker of clinical response in CM patients	Conroy et al., 2010
Ligands of chemokine receptor CXCR3	CXCL4, CXCL10	CSF, serum	ELISA based assays	Enhanced plasma levels of CXCL10 and CXCL4 are significantly associated with a poor outcome in CM and could be used to determine mortality risk in patients	Wilson et al., 2011
Soluble ICAM-1	sICAM-1	Endothelial (plasma levels)	ELISA based assays	High levels of soluble ICAM-1 are strongly associated with CM	Adukpo et al., 2013
Specific muscle proteins	CA3 (major), CK, CKM, MB	Vascular (plasma levels)	Affinity-Proteomics	Elevated levels of specific muscle proteins in plasma indicate muscle damage and microvasculature lesions in children with CM	Bachmann et al., 2014
Pf histidine-rich protein-2	PfHRP2	CSF	ELISA based assays	Elevation of CSF pfHRP-2 is indicative of mortality in CM patients	Thakur et al., 2014
**EXPERIMENTAL CEREBRAL MALARIA**					
Endothelin-1	ET-1	Endothelial	RT/qRT-PCR	Significant increase of mRNA levels of ET-1, coding enzyme ECE and its receptors (ET-A, B)	Machado et al., 2006
Chemokine receptor CXCR3 and its ligands (Mig, IP-10)	CXCR3, Mig, IP-10	N/A	qPCR/ELISA	CXCR3 is essential for trafficking of T cells into the brain and the development of ECM. CXCR3 ligands (Mig and IP-10) have distinct and non-redundant roles in ECM pathogenesis	Campanella et al., 2008
Glutamate	Glu	Brain	Enzyme assays, SHIRPA screen	Increased levels of glutamate leads to CNS dysfunction, neurological and behavioral symptoms	Miranda et al., 2010
Cerebral levels of IL-1β and TNF	IL-1β and TNF	CSF	ELISA based assays	Increased cerebral levels of IL-1β and TNF are associated with anxiety-like behavior	Miranda et al., 2010
CD8+ T Cells and IFN-γ	CD8+ T cells, IFN-γ	NA	ELISA based assays	CD8+ T Cells and IFN-gamma are required for time-dependent accumulation of PRBC in deep organs	Claser et al., 2011
Granzyme B expression	GzmB	CD8+ T cells	*gzmB*^−∕−^ knock out mice	High expression of Granzyme B on CD8+ T cells reduces parasite biomass in the brain; ECM induction is dependent on antiparasitic CD4+ T cell responses	Haque et al., 2011
Platelet Activating Factor	PAF	Endothelial	PAFR^−∕−^ knock out mice	Facilitates the recruitment of leukocytes, induces the release of immune factors; increases vascular permeability	Lacerda-Queiroz et al., 2012
Brain water channel aquaporin-4	AQP4	CSF	Semi-quantitative RT-PCR	Partial protection conferred by AQP4	Promeneur et al., 2013
Plasma microparticles	MP	Blood	Biochemical	Mediates coagulation, inflammation, and cell adhesion; facilitates neurological lesions	El-Assaad et al., 2014b

*Epo, erythropoietin; PfEMP-1, Plasmodium falciparum Erythrocyte Membrane Protein-1; AQP4, Aquaporin 4; ECM, experimental CM*.

### Early screening and diagnosis biomarkers

**Figure 1:**
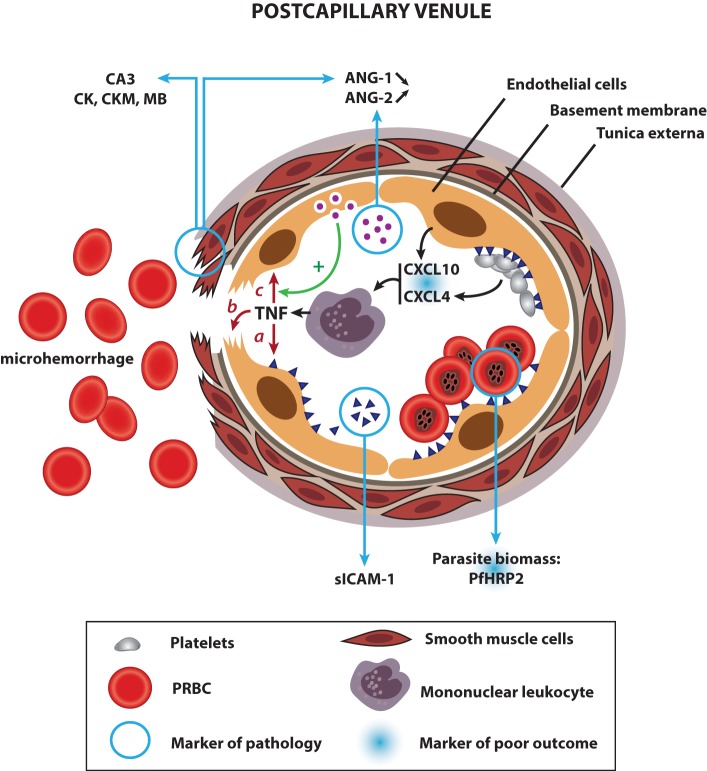
**Links between human CM physiopathology and current biomarkers of pathogenesis and poor outcome: a proposed model**. Upon infection with *P. falciparum*, the host immune system produces pro-inflammatory cytokines, which activate endothelial cells, prompting them to produce CXCL10, a chemoattractant for mononuclear leukocytes. Platelets accumulated in the microvasculature of CM patients release CXCL4 from their alpha granules, which stimulates the production of TNF by mononuclear leukocytes locally. Both cytokines contribute to a hyperinflammatory state in CM and are associated with a poor outcome. Once released, TNF leads to the upregulation of ICAM-1 on endothelial cells (a), which, in turn, induces the sequestration of PRBC and platelets in the cerebral microvasculature. The presence of soluble ICAM-1 in the plasma is reflective of the increase in ICAM-1 levels at the endothelial surface. High densities of sequestered parasites produce elevated amounts of PfHRP2, a marker of pathology and an indicator of poor outcome when detected in the CSF. Sequestration of parasites, coupled with high levels of TNF induce focal vascular injuries, leading to smooth muscle cell damage and ring hemorrhages (b). Injured smooth muscle cells discharge abnormally high amounts of carbonic anhydrase III (CA3), creatine kinase (CK), creatine kinase, muscle (CKM), and myoglobin (MB) in the bloodstream, all biomarkers of CM. In addition, destroyed smooth muscle cells stop producing ANG-1, contributing to its systemic decrease. Coupled with an elevated release of ANG-2 from the Weibel–Palade bodies of activated endothelial cells, the shift in the angiogenic factor balance results in a high ANG-2:ANG-1 ratio, another marker of pathology. ANG-2 sensitizes endothelial cells, which become responsive to sub-threshold concentrations of TNF, contributing to the aggravation of the different pathways described above (c).

Biomarkers used for early screening or diagnosis are used as an indicator of a biological factor that represents either a subclinical manifestation, stage of the disorder, or a surrogate manifestation of the disease. Early screening biomarkers allow the identification of individuals destined to become affected or who are in the “preclinical” stages of the illness. Unfortunately, due to the rapid development of CM and the late presentation of the patients to hospitals, longitudinal analyses of plasma from patients with falciparum malaria have not been feasible so far and potential early screening biomarkers for CM before the onset of symptoms are yet to be identified. However, serological factors that allow the accurate discrimination of CM after the onset of symptoms have been described in the recent years. These biomarkers are indicative of pathology, as they are based on specific processes that have been associated with the development of CM (Figure 1). For instance, the role of endothelial intra-cellular adhesion molecule-1 (ICAM-1) in the sequestration of PRBC is well-understood, and specific binding of PRBC to ICAM-1 has been implicated in the development of CM (Smith et al., 2000). High levels of plasma soluble ICAM-1 were found to be associated with the development of CM in Ghanaian children, and these levels may reflect the upregulation of ICAM-1 in the cerebral microvasculature (Adukpo et al., 2013). Angiopoetin-1 and -2 (ANG-1 and -2) are critical regulators of endothelial activation and integrity, and their levels have also been described as reliable biomarkers of CM. Indeed, ANG-1 and -2 levels profiled from serum or whole blood were shown to discriminate accurately between cerebral and uncomplicated malaria in African patients (Lovegrove et al., 2009), and between cerebral, severe non-cerebral malaria, and uncomplicated malaria in a cohort of Thai patients (Conroy et al., 2009). Compared to UM, CM patients presented significant decreases in ANG-1 and increases in ANG-2 levels and the ratio of ANG-2:ANG-1. This is consistent with the pathophysiology of CM, which involves endothelial activation and dysfunction. Indeed, ANG-1 maintains vascular quiescence, while ANG-2 displaces ANG-1 upon endothelial activation and sensitizes the cells to become responsive to sub-threshold concentrations of tumor necrosis factor (TNF; Kim et al., 2011). Estimation of *Plasmodium falciparum* histidine rich protein 2 (PfHRP2) in the plasma samples has also been shown to be an accurate diagnostic tool to ascertain the parasite biomass in severe malaria patients, and allowed the distinction between severe and uncomplicated malaria (Hendriksen et al., 2012; Imwong et al., 2015). Clinical studies have also shown that PfHRP2 can be present in the CSF of patients with CM (Mikita et al., 2014). More recently, the use of advanced affinity-proteomic tools employing a high-throughput platform of specific antibodies for candidate screening has allowed a wider analysis of potential diagnosis biomarkers for the neuropathology. The plasma levels of 1015 muscle proteins were measured in 700 children and Bachmann and colleagues showed that high levels of four specific smooth muscle proteins exhibit high correlation with the development of endothelial injury and microvasculature lesions during CM, including the smooth muscle cells that surround the endothelial cell monolayer in the *tunica media* of post-capillary venules (Bachmann et al., 2014). This is in concordance with the presence of vascular and microvascular lesions complicated by ring hemorrhages in comatose CM patients (Ponsford et al., 2012). In addition, as ANG-1 is primarily produced by vascular smooth muscle cells (van Meurs et al., 2009), their injury could result in a reduced production of the angiogenic factor, leading to its low levels during CM. Further studies are warranted but these proteins could represent new biomarkers for the severity of CM and allow prompt therapeutic measures.

### Prognostic biomarkers

Prognostic biomarkers provide information on the likely course of the disease in an individual. Plasma levels of ANG-1 and ANG-2 can also predict the clinical outcome of CM, according to studies performed in African children (Lovegrove et al., 2009) and in Indian adults (Jain et al., 2011), in which low ANG-1 levels on presentation was associated with a fatal outcome. This may indicate that, in addition to antiparasitic drugs, ANG-1 is needed to reverse the deleterious endothelial activation in CM and prevent death (Wassmer et al., 2015). CXCL10 and CXCL4 (C-X-C motif chemokine 10 and 4), the ligands of chemokine receptor CXCR3, were described as another set of prognostic biomarkers in CM (Wilson et al., 2011). Indeed, high plasma levels of the chemokines were found to be significantly associated with mortality in CM patients. CXCL10 is produced by a variety of cells, including endothelial cells. The effects of elevated levels of CXCL10 on the cerebral microvasculature are unknown but are suspected to cause local injuries by recruiting mononuclear leukocytes, inducing focal hyperinflammation (Jain et al., 2008). In addition, CXCL4 is released from activated platelets and stimulates TNF production by mononuclear leukocytes, a key proinflammatory cytokine associated with the development of CM. This study established that CXCL10 and CXCL4 can be routinely used to predict the mortality risk in CM patients in endemic settings. Along with the clinical diagnosis of CM, the presence of PfHRP2 in CSF can also be used to predict the disease outcome, as recently demonstrated in retinopathy positive CM patients from Malawi. Using 100 CSF and 103 plasma samples, their findings inferred that an increased level of PfHRP2 in CSF and lower plasma/CSF PfHRP2 ratios was predictive of death in retinopathy positive CM patients (Thakur et al., 2014).

### Future directions and market barriers

Host plasma microparticles (MP) are submicron elements (100 nm–1 μM), which originate from extracellular vesiculation processes during host cell activation and/or apoptotic events. High MP numbers were shown to be significantly higher in the plasma of patients with CM compared to patients with uncomplicated malaria or severe anemia in several separate analyses (Combes et al., 2004; Pankoui Mfonkeu et al., 2010; Nantakomol et al., 2011), showing that they could be potentially used as a diagnosis biomarker for CM. In addition, platelet-derived MP were shown to be the most abundant in the plasma of CM patients, and their levels were significantly correlated with coma depth and thrombocytopenia. However, the current analysis of plasma MP necessitate high-sensitivity clinical equipment and trained technicians, which might explain why their use as a biomarker in endemic regions has not been further investigated to date. The fast-paced evolution of low-cost, portable, point-of care quantitative diagnostic devices might reverse the situation in the near future. Similarly, cumulating data suggest that small non-coding-RNAs such as microRNAs (miRNAs) can be utilized as potential biomarkers for the diagnosis and prognosis of a variety of parasitic diseases (Manzano-Roman and Siles-Lucas, 2012). Circulating miRNAs can be detected in biological fluids as serum, saliva and others, exhibiting a good potential as non-invasive biomarkers. While this has not yet been evaluated in falciparum malaria infection, it is likely that the current technology required for miRNA identification and quantification will restrict their use as diagnostic or prognosis biomarker for now. Lastly, In addition to biomarkers from biological media, the recent implementation of MRI techniques to investigate the factors leading to the development of CM might also lead to the identification of biomarkers of severity and/or disease outcome using imaging maps. Such approaches could focus on the parasite burden in the brain and correlate it with disease severity, or establish a scale of brain swelling in CM patients, potentially indicative of the disease prognosis.

## Emerging therapeutic options

The major challenge to prevent human mortality in CM lies in the current lack of specific therapies aimed at dampening the pro-inflammatory state associated with the neurologic syndrome, as well as its deleterious effects on the host. Considering the multi-factorial nature of the neuropathology, even the most effective anti malarial drugs cannot ensure complete survival (Dondorp et al., 2005, 2010; Mishra and Wiese, 2009) and due to the poor understanding of its pathogenic processes, candidate adjunctive therapies to decrease mortality in CM have been unsuccessful so far (Mohanty et al., 2006, 2011; Mishra and Wiese, 2009; John et al., 2010). However, the recent breakthrough allowed by some of the technologies and approaches described above are slowly narrowing the spectrum of candidate therapeutic pathways. Table 2 represents some of the potential therapeutics against CM, based on recent observations from experimental models and human pathogenesis. Some of these novel adjunct therapies include heme-oxygenase-1 (HO-1) and carbon monoxide (CO), exogenous nitric oxide, pressurized oxygen, additive antioxidants, and hydrogen sulfide gas (Dellavalle et al., 2013; Table 2).

**Table 2:** **Cerebral malaria: emerging therapeutic options**.

**Therapeutics**	**Mode**	**Observation/mechanism of protection**	**References**	**Stage**
**HUMAN CEREBRAL MALARIA**				
Levetiracetam (LVT1)	Single	Treatment of seizures, epilepsy and improves CM outcome in African children	http://www.clinicaltrials.gov/ct2/show/study/NCT01660672	Clinical trial completed, Oct 2014
Rosiglitazone	AP-partnered	Decreases levels of pro-inflammatory factors (IL-6 and MCP-1); elevates BDNF levels (day 2) and lowers Ang-2/Ang-1 ratio (day 3)	Serghides et al., 2014	*In vivo* study
**EXPERIMENTAL CEREBRAL MALARIA**				
Heme-Oxygenase-1 (HO-1) and carbon monoxide (CO)	Adjunctive	Decreases parasitemia, prevents BBB disruption, brain microvasculature congestion, neuro-inflammation and CD8+ T-cell brain sequestration during ECM	Pamplona et al., 2007	Experimental
Pressurized oxygen (HBO) therapy	Adjunctive	Prevents ECM signs; reduces expression of TNF, IFN-γ and IL-10 mRNA levels and percentage of γδ and αβ CD4+ and CD8+ T cell sequestration, prevents BBB dysfunction	Blanco et al., 2008	Experimental
Thiol Pantethine	Adjunctive	Decreases circulating microparticles and protects BBB integrity	Penet et al., 2008	Experimental
Nimodipine	Artemether-partnered	Prevents vasoconstriction and vascular collapse by inducing vasodilation and enhancing pial blood flow; increases survival	Cabrales et al., 2010	Experimental
Artemisone	CQ-partnered	Prevents mortality at late stages of ECM	Waknine-Grinberg et al., 2010	Experimental
Antioxidant therapy	CQ-partnered	Prevents the development of persistent cognitive damage	Reis et al., 2010	Experimental
Flt3 ligand	Adjunctive	Helps reducing the proportion of CD8-T cells producing IFN-γ and granzyme B; decreases sequestration of PRBC	Tamura et al., 2011	Experimental
Artemether + Artesunate	Combinative	Prevents death at late-stages of ECM; reduces leukocyte accumulation in brain vessels and decreases cerebral vascular inflammation	Clemmer et al., 2011	Experimental
Exogenous Nitric Oxide (NO)	Single	Leads to a decreased expression of ICAM-1 and P-selectin; a lower number of adherent leukocytes and platelets in pial vessels and in venules; a reduced vascular inflammation, and albumin leakage	Zanini et al., 2011	Experimental
Erythropoietin	Single	Reverses the development of ECM and degree of neural hypoxia; reduces clinical signs of CM and cerebral pathology features	Hempel et al., 2011, 2012	Experimental
HJP-272	Artemether-partnered	Decreases brain hemorrhage and increases survival	Dai et al., 2012	Experimental
IDR-peptide	Adjunctive	Enhances mice survival in late stage interventions through anti-inflammatory networks	Achtman et al., 2012	Experimental
Atorvastatin	Adjunctive	Prevents parasite cytoadherence and endothelial damage; enhances the pharmacologic inhibition of CXCL10 and a reduction in mortality	Taoufiq et al., 2011; Wilson et al., 2011	Experimental
Rosiglitazone	Adjunctive	As an agonist of Peroxisome proliferator-activated receptor-γ (PPAR-γ), modulates host inflammatory responses and improves clinical outcome in ECM; prevents the development of brain atrophy and neurocognitive impairment	Serghides et al., 2009, 2014	Experimental
Neuregulin-1 (NRG-1)	Single; Compared with Artemether	Leads to endothelial protection and reduction in BBB permeability; neuro-protective nature, decreases mortality	Li et al., 2012; Lok et al., 2012; Solomon et al., 2014	Experimental
Citicoline (CTC)	Adjunctive	CTC reduces the production of microparticles *in vitro*; confers protection against ECM	El-Assaad et al., 2014a	Experimental

*BBB, blood-brain barrier; ECM, experimental CM; IDR-peptide, innate defense regulatory peptide; CQ, Chloroquine; AP, Atovaquone–Proguanil; BDNF, brain-derived neurotrophic factor*.

In addition to adjunct therapies, new classes of agents developed using novel creative strategies are urgently needed to tackle severe malaria infection. Indeed, the number of available and effective antimalarial drugs is quickly dwindling, as the resistance of *P. falciparum* against artemisinin combination treatments (ACT), the recommended first-line therapy for infected patients, is now prevalent across mainland Southeast Asia (Ashley et al., 2014). This is alarming because: (i) resistance to the previous mainstays of antimalarial treatments—namely chloroquine and sulfadoxine/pyrimethamine—have already spread across southeast Asia into Africa, resulting in the deaths of millions of patients (Roper et al., 2004) and (ii) there are currently no alternative drugs to replace ACT. However, unprecedented global and multidisciplinary efforts aimed at broadening therapeutic potential and identifying novel modes of action are currently in place (Flannery et al., 2013). Hopefully, these efforts will allow the widening of malaria treatement options and will help to overcome the emerging drug resistance.

## Concluding remarks

The World Health Organization estimated that deaths related to malaria have dropped down from one million in 2000 to about 780,000 in 2009 and 627,000 in 2013 (W.H.O., 2011, 2014). In endemic Africa, unprecedented levels of intervention coverage have halved *Plasmodium falciparum* infection prevalence since 2000, and the incidence of clinical disease have dropped by 40% (Bhatt et al., 2015). Despite this promising trend, the figures are still worrisome. Such progress has been possible despite the lack of a malaria vaccine and is mainly attributable to increased prevention efforts globally, coupled to the widespread use of artemesinin derivatives. The systematic use of some of the emerging new technologies aimed at facilitating the early and accurate diagnosis of malaria-associated complications is likely to not only help clinicians identify high-risk CM patients in the future, but also expedite the clinical triage of symptomatic parasitemic patients, as well as their prompt and comprehensive care. This will, hopefully, lead to a further decrease in malaria-related deaths worldwide. In addition, the recent implementation of state-of-the-art investigation tools to elucidate the pathophysiology of CM is also likely to contribute to the identification of new image-derived prognosis biomarkers and adjunct therapeutic avenues, as illustrated by the recent and groundbreaking study of brain lesions in Malawian patients with CM (Seydel et al., 2015). Lastly, the European Medicines Agency just approved the RTS,S vaccine with the recommendation that it should be used in African children at risk, the first malaria vaccine to ever get this clearance (Hawkes, 2015). Coupled with the clinical efforts in malaria-endemic regions, this vaccine could represent a milestone in the global fight against malaria.

## Funding

This work was supported by the National Institute of Allergy and Infectious Diseases, National Institutes of Health, grant U19AI089676-01S1 (to SCW). The content is solely the responsibility of the authors and does not necessarily represent the official views of the National Institutes of Health.

### Conflict of interest statement

The authors declare that the research was conducted in the absence of any commercial or financial relationships that could be construed as a potential conflict of interest.
